# A G_o_-type opsin mediates the shadow reflex in the annelid *Platynereis dumerilii*

**DOI:** 10.1186/s12915-018-0505-8

**Published:** 2018-04-18

**Authors:** Thomas Ayers, Hisao Tsukamoto, Martin Gühmann, Vinoth Babu Veedin Rajan, Kristin Tessmar-Raible

**Affiliations:** 1 Max F. Perutz Laboratories, University of Vienna, Campus Vienna Biocenter, Dr. Bohr-Gasse 9/4, 1030 Vienna, Austria; 2 Department of Life and Coordination-Complex Molecular Science, Institute for Molecular Science, Okazaki, 444-8585 Japan; 3 Department of Structural Molecular Science, SOKENDAI (The Graduate University for Advanced Studies), Hayama, Kanagawa, 240-0193 Japan; 4 Japan Science and Technology Agency (JST), Precursory Research for Embryonic Science and Technology (PRESTO), Kawaguchi, Saitama, 332-0012 Japan; 5 Max Planck Institute for Developmental Biology, Paul-Ehrlich Straße 20, 72076 Tübingen, Germany; 6 Research Platform ‘Rhythms of Life’, University of Vienna, 1030 Vienna, Austria

**Keywords:** behaviour, opsins, non-visual, marine, *Platynereis dumerilii*, annelid, shadow reflex, peripheral photoreceptors, evolution

## Abstract

**Background:**

The presence of photoreceptive molecules outside the eye is widespread among animals, yet their functions in the periphery are less well understood. Marine organisms, such as annelid worms, exhibit a ‘shadow reflex’, a defensive withdrawal behaviour triggered by a decrease in illumination. Herein, we examine the cellular and molecular underpinnings of this response, identifying a role for a photoreceptor molecule of the G_o_-opsin class in the shadow response of the marine bristle worm *Platynereis dumerilii.*

**Results:**

We found *Pdu-Go-opsin1* expression in single specialised cells located in adult *Platynereis* head and trunk appendages, known as cirri. Using gene knock-out technology and ablation approaches, we show that the presence of *Go-opsin1* and the cirri is necessary for the shadow reflex. Consistently, quantification of the shadow reflex reveals a chromatic dependence upon light of approximately 500 nm in wavelength, matching the photoexcitation characteristics of the *Platynereis* Go-opsin1. However, the loss of *Go-opsin1* does not abolish the shadow reflex completely, suggesting the existence of a compensatory mechanism, possibly acting through a ciliary-type opsin, *Pdu-*c-opsin2, with a Lambda_max_ of approximately 490 nm.

**Conclusions:**

We show that a *Go-opsin* is necessary for the shadow reflex in a marine annelid, describing a functional example for a peripherally expressed photoreceptor, and suggesting that, in different species, distinct opsins contribute to varying degrees to the shadow reflex.

**Electronic supplementary material:**

The online version of this article (10.1186/s12915-018-0505-8) contains supplementary material, which is available to authorized users.

## Background

The shadow reflex is a defensive withdrawal behaviour shared between many sessile and sedentary organisms. Well-described examples include different species of bivalves, which swiftly close their shell or retract their siphons [1]. Detailed attention has been paid to the spectrum that causes such a response in scallops [2], as well as the possible role of their mantle edge eyes in this response [3, 4]. Fan worms (*Sabellidae* and *Serpulidae*) exhibit a pronounced shadow response, withdrawing into their opaque tubes upon the disillumination of photoreceptors housed in their radiolar tentacles [5–7]. The primary function of these photosensitive structures, and those more recently documented in ark clams, is likely to be shadow detection [6], but so far research into the underlying molecular mechanisms of the shadow reflex has been limited due to the dearth of molecular tools available in these model organisms. *Platynereis dumerilii,* a molecularly slowly evolving [8, 9], morphologically archetypical marine nereidid [10], and distant annelid relative of sabellid fan worms [11–13], with its expansive genetic toolkit [14] and observable, well-characterised shadow reflex [15], represents a useful model to understand the molecular mechanisms and evolution of this behaviour. *Platynereis dumerilii* is a predominantly nocturnal animal, but the animals also exhibit movement and exploratory behaviour during the day, anchoring their trunks inside protective tubes whilst occasionally reaching out with their heads, likely to survey the environment and to feed [16]. Sudden darkness or a passing shadow leads to a highly reproducible longitudinal contraction of the body, which retracts the head and tail within the confines of the tube [15].

The photoreceptor molecules responsible for governing the shadow response are unknown, though two distinct protein subtypes represent potential shadow reflex light receptors. On the one hand, accumulating evidence suggests that a ciliary-type opsin (c-opsin) mediates the shadow reflex of fan worms, and c-opsins exist in *Platynereis dumerilii* [17, 18]. On the other hand, the first member of the *Go-opsin* class (belonging to the animal Tetraopsins group [19]) was identified and characterised in the mantle-edge eyes of the scallop, *Patinopecten yessoensis* [20]. Scallops are known for their distinct shadow reflex [2] and predator avoidance responses, thought to be mediated by their mantle-edge eyes [6, 21]. Go-opsins hence represent good photoreceptor candidates for the shadow response in this group. *Go-opsin1* in *Platynereis* larvae has been shown to be responsible for the phototactic response to light [22], a useful function during their short, free-swimming pelagic stage. *Platynereis* larvae typically settle at the bottom at approximately 1 week of age and spend most of their entire remaining life in and around self-spun tubes. Sudden changes of surrounding conditions, such as light, lead to an immediate retraction of the worm into its home tube. Herein, we aim to quantitatively assess the shadow reflex in *Platynereis dumerilii* and use genetic tools to ascertain which photoreceptive proteins and anatomical regions of the organism are required for this behavioural response.

## Results

### The shadow response in *Platynereis dumerilii* is wavelength dependent and maximally activated by 500 nm light

**Fig. 1:**
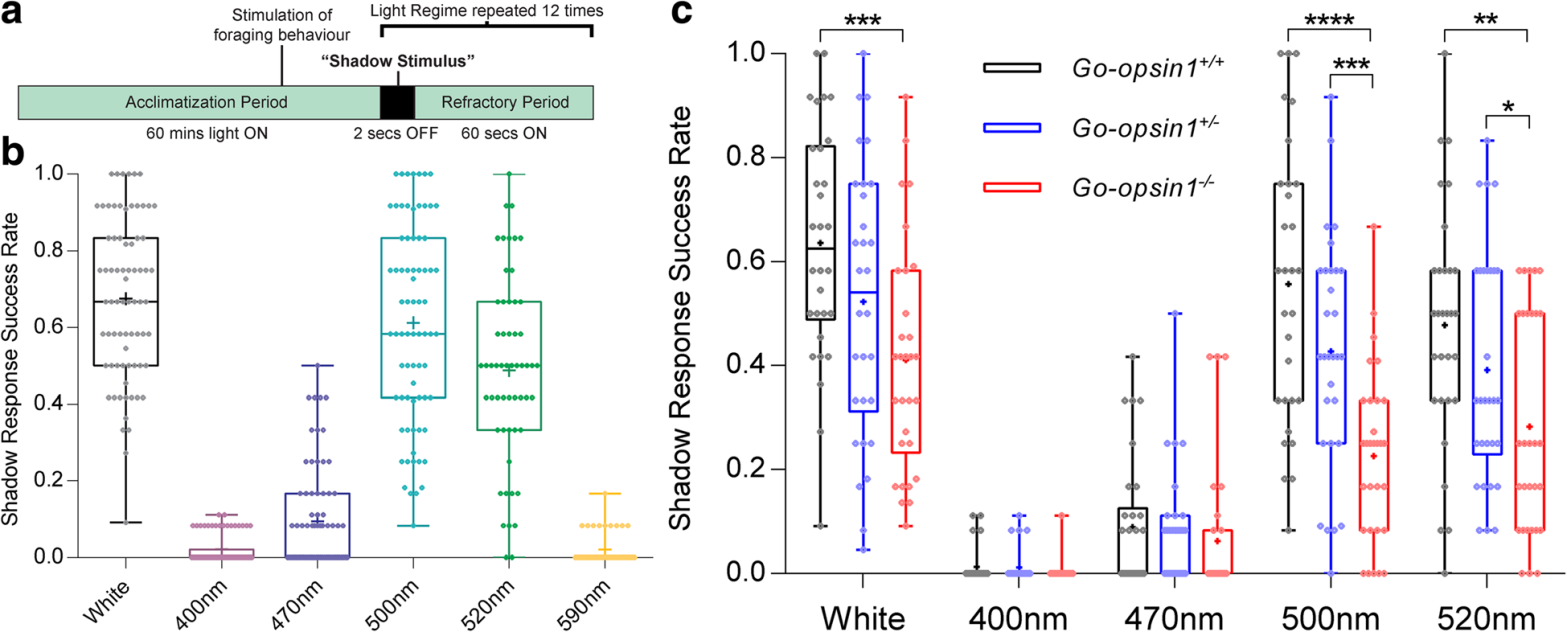
Wavelength dependence of the adult *Platynereis* shadow response and impaired shadow reflex in Go-opsin1^−/−^ animals. **a** Schematic of light regime used for the Shadow Reflex Assay. Following a 60 min acclimatisation period of constant light, 12 ‘shadow stimuli’, consisting of 2 s periods of abrupt darkness, occur between 60 s intervals of light. Stimulation of foraging behaviour refers to the addition of Spinach-conditioned artificial seawater 15 min prior to the first shadow (see also Additional file 1: Movie S1 and Additional file 2). **b** Efficiency of the Shadow Reflex in wildtype adult *Platynereis dumerilii* subjected to shadows under white light (black, *n* = 74) and five different monochromatic light conditions, 400 nm (purple, *n* = 74), 470 nm (blue, *n* = 74), 500 nm (cyan, *n* = 74), 520 nm (green, *n* = 56) and 590 nm (orange, *n* = 44). Whiskers in the box plot indicate the range of scores; means are indicated with a cross whilst individual scores are plotted as diamonds. Data from 7 experimental days are combined. **c** Efficiency of the Shadow Reflex in sibling *Go-opsin1*^+/+^ (black, *n* = 30), *Go-opsin1*^Δ8/+^ (blue, *n* = 30) and *Go-opsin1*^Δ8/Δ8^ (red, *n* = 30) adult *Platynereis dumerilii* under five different light conditions. Whiskers in the box plots indicate the range of scores; means: cross, individual scores: diamonds. Significance scores (** < 0.01, *** < 0.001, **** < 0.0001) represent *P* values of Mann–Whitney U tests comparing wildtype, heterozygous and homozygous siblings


We first established a shadow reflex paradigm assayable under chromatically variable light sources, consisting of a 2 s period of complete darkness (Fig. 1a, Additional files 1 and 2A, B). Under white light, the worms reproducibly perform their recognisable behaviour upon sudden loss of illumination (Fig. 1b). Further analyses using LEDs with different specific maximal emission spectra (‘monochromatic light’; Additional file 2) revealed that the *Platynereis* shadow reflex is wavelength dependent and maximally triggered by approximately 500 nm, or cyan, light (Fig. 1b), with a mean Shadow Response Success Rate (SRSR) comparable to that of white light. We find significantly decreased SRSRs under 520 and 470 nm and a virtually non-existent shadow reflex under 400 and 590 nm light, forming a distinct photoexcitation curve for the response (Fig. 1b). It should be noted that, whilst our 500 , 520 and 590 nm light conditions have comparable intensities (1 × 10^12^ photons/cm^2^/s), the 400 and 470 nm light conditions have an approximately 10-fold greater intensity (3 × 10^13^ photons/cm^2^/s; Additional file 2C, D), but still exhibit strongly decreased shadow responses in wild-type animals (Fig. 1b).

Additional file 1:**Movie S1.** Shadow reflex assay infrared recording showing 35 wild type worms subjected to three subsequent white light shadow stimuli with the refractory period between each shadow included. A white circle in the upper left corner signifies light ON and no white circle signifies light OFF. Immediately upon loss of the light stimulus, animals are graded binarily as described in Methods. For each stimulus, green circles indicate animals scored 1 for a successful response, whilst magenta circles indicate animals scored 0 for no response. Indicator circles appear three seconds prior to loss of light. (MP4 1662 kb)


### *Go-opsin1* is required for the shadow response in adult *Platynereis dumerilii*

**Fig. 2:**
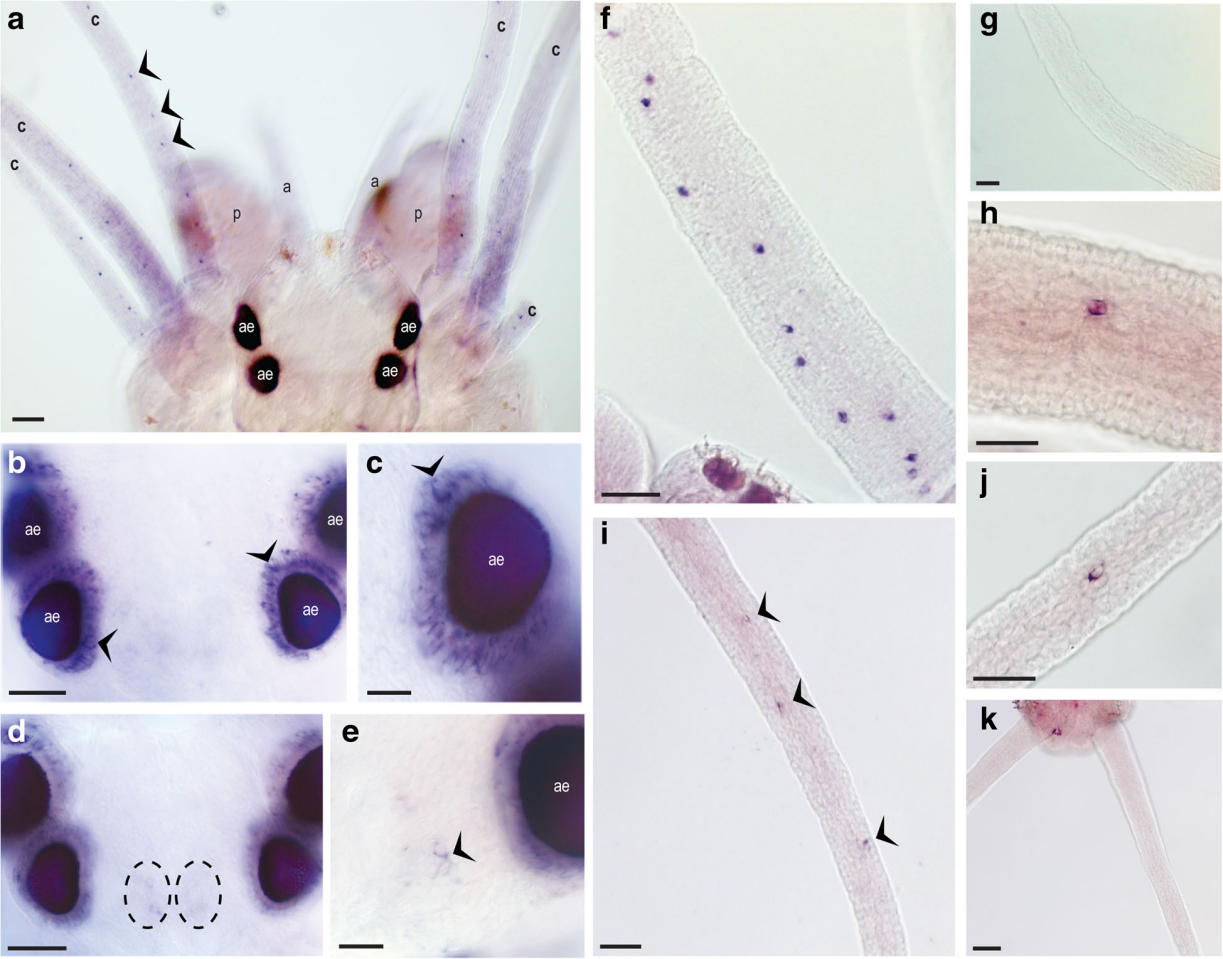
Expression domains of *Go-opsin1* in adult *Platynereis dumerilii.* **a–e** Differential Interference Contrast images of adult *Platynereis dumerilii* (aligned anterior–posterior:top–bottom) stained by whole mount *in situ* hybridisation for *Go-opsin1* (purple) (Additional file 5)*.* **a** Whole head overview with *Go-opsin1* expression in cells aligned along the edge of the peristomal cirri (**c**). Adult eyes (ae), palps (p) and antennae (a) are also indicated. Ocular expression (**b, c**) and dorsal neuronal expression (**d, e**) are shown. 40× magnification of *Go-opsin1* cells in the peristomal cirri (**f**) and anal cirri (**i**) are shown alongside corresponding sense probe negative controls (**g**; peristomal cirri, **k**; anal cirri). 63× magnification of an individual *Go-opsin1*-expressing cell is shown in the peristomal (**h**) and anal (**j**) cirri. Scale bars: 50 μm for a, b, d, i and k, and 20 μm for c, e, f, g, h and j. Black arrowheads: *Go-opsin* expressing cells

The spectral dependence of the *Platynereis* shadow response (Fig. 1b) matches closely with the action spectrum of *Pdu-*Go-opsin1, whose Lambda_max_ is approximately 498 nm [22], and we show that *Go-opsin1* expression indeed persists in adult *Platynereis* (Fig. 2). Considering that adult *Platynereis* are predominantly benthic organisms and thus rarely engage in phototactic swimming behaviours, the role of Go-opsin1 in adult worms has remained enigmatic. We made use of worms carrying a previously characterised mutation in the *Pdu-Go-opsin1* gene, which results in a premature stop codon and likely complete loss-of-function [22]. We tested homozygous mutant worms against heterozygous mutants and sibling wildtypes in our shadow response paradigm, and found that the loss of *Pdu-Go-opsin1* significantly reduces the SRSR under white light (*P* < 0.0003), 500 nm light (*P* < 0.0001) and 520 nm light (*P* < 0.0036) (Fig. 1c), demonstrating Go-opsin1 dependency. Our data also suggests a Go-opsin1 protein level dependence, as the mean SRSRs of *Go-opsin1*^*+/−*^ animals fall between those of their wildtype and homozygous siblings under white, 500 nm and 520 nm light (Fig. 1c), emulating the protein level dependence of the phototactic response present in *Platynereis* larvae [22].

Besides the shadow response, we also re-evaluated the circalunar maturation timing of mutant worms versus wildtype siblings under monochromatic 500 nm light. *Go-opsin*^+/−^ or ^−/−^ animals exhibited a normal circalunar maturation rhythm compared to their wildtype siblings (Additional file 3).

### Cirri of adult *Platynereis dumerilii* express *Go-opsin1* and are required for the shadow response

We next assessed via which cells *Go-opsin1* mediates its shadow reflex function. Expression in *Platynereis dumerilii* larvae is limited to the larval and adult eyes and a neuron proximal to the ciliary photoreceptor cells of the brain [22]. We confirmed expression in the adult eyes and found an expanded domain proximal to the ciliary photoreceptors in the adult animals (Fig. 2b–e). We also identified novel expression domains in the adult worm’s peripheral structures, known as cirri (Fig. 2a, f–k). Eight peristomal cirri branch out from the lateral edge of the prostomium at the anterior end of the worm and two anal cirri extend from the posterior final segment. All 10 of these cirri possess *Pdu-Go-opsin1*-expressing single cells (Fig. 2a, f–k).

**Fig. 3:**
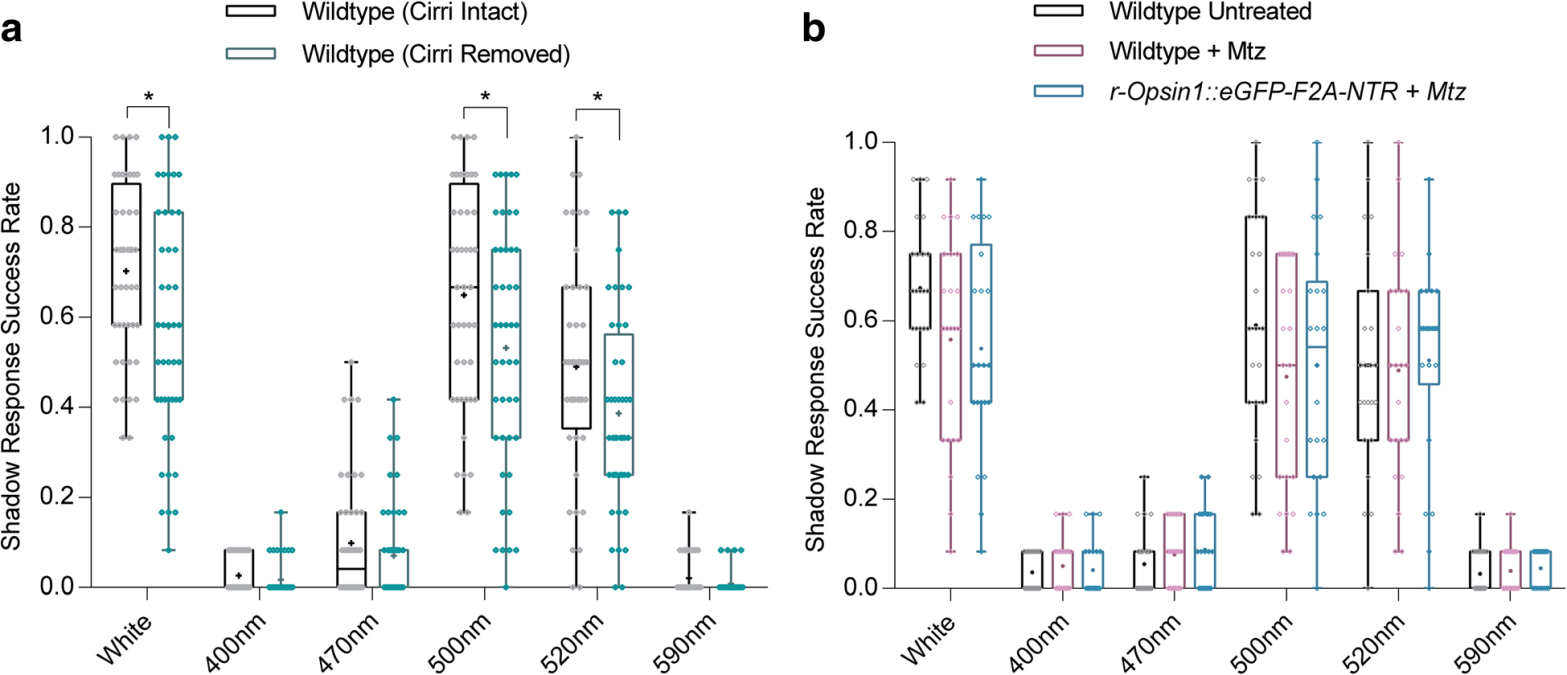
Requirement of the *Platynereis* shadow reflex on cirral extremities, but not rhabdomeric photoreceptor cells. **a** Shadow Response Success Rate (SRSR) of wildtype (black, *n* = 44) and sibling wildtype animals with peristomal and anal cirri removed (green, *n* = 44) under six different light conditions. Whiskers in the box plot indicate the range of scores; means are indicated with a cross whilst individual scores are plotted as diamonds. Significance scores (* < 0.05) of *P* values of Mann–Whitney U tests are displayed above boxes comparing Cirri^+^ and Cirri^–^ animals (see also Additional file 4). **b** SRSR of untreated wildtype worms (black, *n* = 23), wildtype animals (purple, *n* = 23) and *r-opsin1::egfp-f2a-ntr* transgenic worms (cyan, *n* = 22) treated with 12 mM of metronidazole in 0.02% DMSO under six different light conditions. Whiskers in the box plot indicate the range of scores; means are indicated with a cross whilst individual scores are plotted as diamonds. No significant differences were found between tested groups, as calculated according to whether the difference between medians (calculated using Hodges–Lehmann non-parametric estimator) exceeded 95% confidence intervals

To assess whether the *Platynereis* cirri are required for the worms shadow response we assayed the shadow reflex efficacy of adult animals whose cirri (both anal and peristomal) had been surgically removed (Additional file 4). Our data show that cirri removal results in significantly decreased SRSRs under white (*P* = 0.023), 500 nm (*P* = 0.0467) and 520 nm (*P* = 0.0227) light (Fig. 3a), indicating that the cirri play an integral role in *Platynereis* shadow detection, likely via *Go-opsin1*-expressing photoreceptive cells.

### The *r-opsin1*-expressing cells of adult *Platynereis dumerilii* do not mediate the shadow reflex

Our data show that neither *Go-opsin1* mutation nor removal of cirri leads to a complete loss of the shadow reflex (Figs. 1c and 3a). These findings strongly suggest that other photoreceptor(s) can compensate for *Go-opsin1* and the loss of cirri. *Pdu-r-opsin1* is a rhabdomeric *opsin*, prominently expressed in the worm’s eyes, as well as in several peripheral trunk photoreceptors [23]. We previously devised a method to specifically ablate all *r-opsin1*^*+*^ cells in a stable transgenic line expressing *gfp* and the bacterial enzyme *nitroreductase* under the *r-opsin1* enhancer [24]. Given that *r-opsin1* is prominently expressed in the rhabdomeric photoreceptors of the worm’s eyes, we reason that this ablation leads to dysfunctional eyes [23]. The complete absence of GFP fluorescence in treated transgenic worms was taken as an indicator for successful *r-opsin1*^*+*^ cell ablation. A higher variability of the SRSR (Fig. 3b) can be seen in metronidazole (mtz)-treated animals, but this is caused by the treatment itself as we find no significant difference between the SRSRs of *r-opsin1::eGFP-f2a-ntr* transgenic animals and equally treated wildtype controls (Fig. 3b), demonstrating that *r-opsin1*^+^ cells are not integral to the *Platynereis* shadow reflex.

### *Pdu*-c-opsin2 excitation characteristics make it a potential secondary shadow photoreceptor

**Fig. 4:**
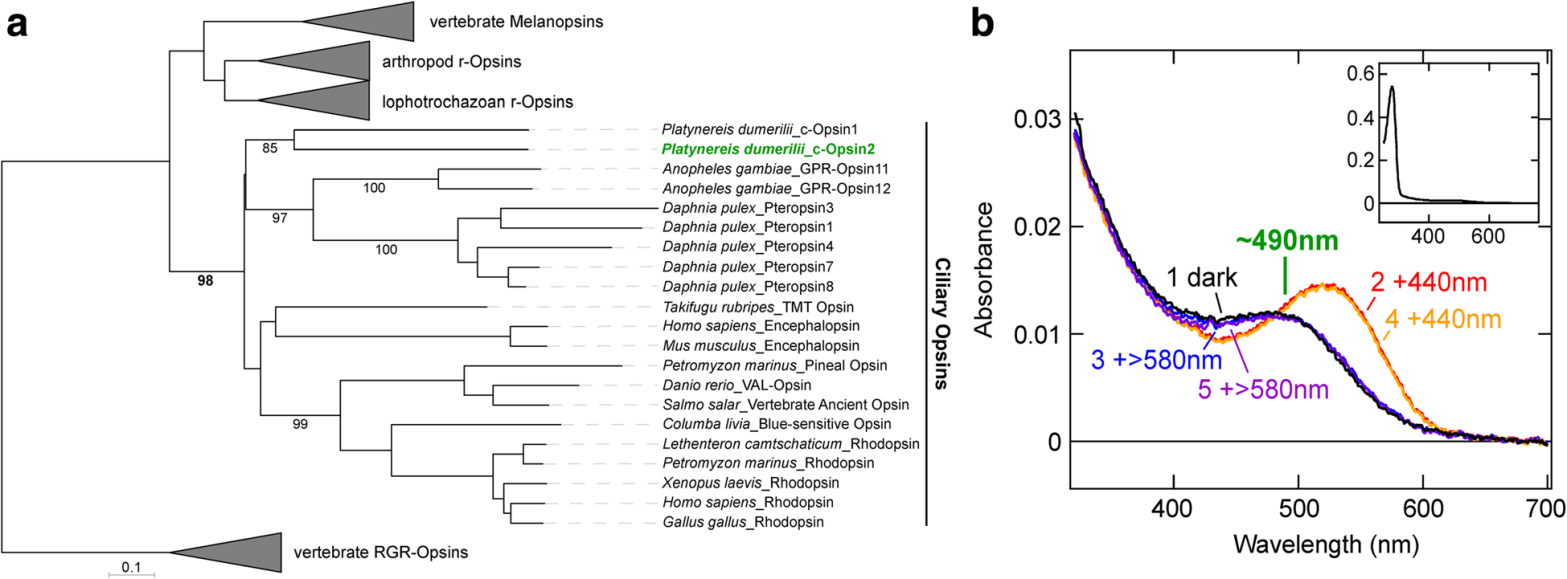
Phylogeny and absorption spectrum of *Platynereis* c-opsin2. **a** Phylogenetic tree showing *Platynereis* c-opsin2 grouped with other ciliary opsins (see also Additional file 6). Bootstrap values are shown in percent (of 1000 bootstraps), bootstraps less than 50 and not pertaining directly to c-opsin2 are omitted. **b** Absorption spectrum of *Platynereis* c-opsin2 purified from COS-1 cells after incubation with 11-cis-retinal (black (1)). Spectra after repeated stimulation for 1 min by 440 nm light (red (2), yellow (4)) and for 30 s at > 580 nm (blue (3), purple (5)) are also shown as traces. The absorption maximum at ~490 nm is highlighted in green. (Inset) The absorption spectrum of *Platynereis* c-opsin2 in a wider wavelength range. Robust absorbance at ~280 nm is attributed to misfolded protein species

Our data indicate that another photoreceptor mediates shadow detection in addition to Go-opsin1, but that the known rhabdomeric photoreceptors of *Platynereis* can be excluded from this function (Fig. 3b). We therefore wondered if a ciliary-type opsin could be a suitable additional candidate, as a c-opsin is suggested to mediate the shadow reflex in fan worms [18]. Two c-opsins exist in *Platynereis*, c-opsin1 and c-opsin2 (please note that we refer to the originally identified *Platynereis* c-opsin [17], as c-opsin1, and to the additionally identified c-opsin as *Pdu*-c-opsin2, while a reversed nomenclature was used in Bok et al. [7]; Fig. 4a). *Pdu*-c-opsin1, is maximally photo-activated between 380 and 400 nm [25], and thus can be discounted from shadow detection due to a negligible wildtype shadow response at 400 nm light (Fig. 1b). However, a second *Platynereis ciliary opsin*, *Pdu-c-opsin2,* is present in head transcriptome data (Fig. 4a) [26]. No other *c-opsins* were identifiable from the worm’s transcriptome data nor genome traces. Sequences of *opsins* from other opsin-families exist, but they do not belong to any of the classes discussed for a role in the shadow reflex, and further expression or spectral characterisation has not been perfromed. We demonstrate here that *Pdu*-c-opsin2 exhibits a maximum absorption wavelength of 490 nm (Fig. 4b), making it a candidate for a secondary shadow detecting photoreceptor. Our data also show that *Pdu*-c-opsin2 is bistable, requiring > 580 nm light to revert to its approximately 490 nm activatable state (Fig. 4b). This quality would also allow the animal to respond relatively continuously to repeated stimuli.

## Discussion

The 2 s-long shadow stimulus of our reflex paradigm is produced by removing the connector plug from the illuminating LED circuit, thereby discontinuing the power (Additional file 1: Movie 1). Although this sudden cessation of light is not identical to a true moving shadow front generated by an object passing between a light source and the point of detection, this paradigm follows shadow reflex paradigms used for vertebrates and crustaceans [27], and a classical paradigm for *Platynereis dumerilii* [15]. The simple cessation of illumination is well reproducible and avoids any potentially startling vibrations, which may arise from passing physical objects above the worms.

The cyan range of light encompasses the wavelengths that typically penetrate water the deepest in coastal areas [28]. The maximal sensitivity of the *Platynereis* shadow reflex in this wavelength range is thus a highly valuable aspect of such a response, which is often crucial for survival.

*Go-opsin1*^+^ cells in *Platynereis* cirri (Fig. 2f–k), the mantle edge eyes of scallops [1] and radiolar eyes and ocelli of fan worms [6] are all positioned at the most distal extent of available extremities. Such peripheral placement of these photoreceptive structures is likely integral to the reliable detection of shadows, even if they do not pass directly over the cephalic or central region of the animal. The photoreceptive cells in the tentacular structures of both serpulids and sabellids have been thoroughly documented [7, 13], and might appear to be more elaborated versions of the simple cirral *Go-opsin1*^*+*^ photoreceptors in *Platynereis dumerilii*. However, several differences are apparent. Firstly, developmental data suggest that the anterior appendages of serpulids and sabellids, the radiolar tentacles, are developmentally homologous to *Platynereis* palpae and not the peristomal cirri [29]. Secondly, the light-sensitive structures of serpulids and sabellids are typically associated with pigment cells [13], which we do not observe in *Platynereis* (Fig. 2a, f–k). Finally, evidence so far points to the involvement of a ciliary-type opsin in fan worm peripheral photodetection [18]. Taken together, these findings suggest that the biological mechanisms conducting the shadow reflexes in *Platynereis* and fan worms have separate evolutionary origins. *Pdu-c-opsin2* and *Pdu-copsin1* can be detected in *Platynereis* head transcriptome data [26]; however, the expression levels of both genes are very low and, despite multiple attempts, were impossible to detect in adult *Platynereis* with the current whole mount *in situ* hybridisation technology.

*Go-opsin1* is co-expressed in the *r-opsin1*^+^ cells within the adult eyes of *Platynereis* (Fig. 2b, c) [22]. Neither these *r-opsin1*^+^ cells in the eyes, nor the non-cephalic *r-opsin1* cells in the parapodia [23] are necessary for the shadow reflex (Fig. 3b). This is congruous with research on sabellid worms whose rhabdomeric-type cephalic eyes, likely using rhabdomeric-type opsins, are suggested to play no part in shadow detection due to their occlusion within the animal’s opaque protective tube [13].

## Conclusions

To conclude, our data strongly suggest that Go-opsin1 functions as one photoreceptor mediating the *Platynereis* shadow reflex, likely via its expression in single cells in the worm’s cirri. A second opsin, c-opsin2, possesses wavelength characteristics that would allow it to function as an additional receptor conducting this crucial and widely conserved survival reflex in *Platynereis*.

## Methods

### Experimental model and subject details

#### *Platynereis dumerilii*

All animals were bred and raised in the Marine Facility at the Max F. Perutz Laboratories in accordance with established protocols [30]. Worm cultures are maintained at 18 °C and a 16 h:8 h light:dark cycle. All wildtype animals used in all shadow assays presented here are of the PIN619512 strain background [14]. *Platynereis dumerilii* worms with an Δ8 base pair deletion in the *Go-opsin1* gene were provided by the laboratory of Gaspar Jekely [22]. In order to obtain wildtype siblings for further analyses, these animals were outbred with the PIN619512 inbred line. Resulting heterozygous worms were subsequently incrossed, resulting in offspring with *Go-opsin1*^*+/+*^*, Go-opsin1*^*+/−*^ *and Go-opsin1*^*−/−*^ genotypes. Worms were genotyped by genomic DNA extraction from a single segment of clipped tail, which was then PCR amplified using standard primer pairs in exon 1 of *Go-opsin1* (Additional file 5), given time to regenerate and subsequently used in the shadow response assay. Animals containing the transgene *r-opsin1::egfp-f2a-ntr* had been generated previously [23]. *Platynereis* animals used in the shadow reflex assay were at the immature stage of development, and therefore cannot be assessed for sex until later stages when sexual differentiation has occurred.

#### COS-1 mammalian cells

COS-1 cell lines were maintained at the Institute for Molecular Science, Okazaki, under standard conditions initially established by G. Khorana [31]. The cell line has not undergone authentication, but has been under continual maintenance by the Khorana, Farrens and Tsukamoto laboratories for approximately 30 years.

### Method details

#### Light condition measurements

The behavioural chamber in which the shadow assay took place was a light-tight insulated box (60 cm × 60 cm × 80 cm) placed in a temperature controlled room (18 °C) on 2-cm thick dampening rubber mats to minimise vibrations. White light conditions were installed in this as described previously [16]. LED arrays (Winger Electronics GmbH & Co.KG) of additional monochromatic wavelengths were constructed and installed in the same configuration at the same distance from the sample, which lies 60 cm from the nearest LED (Additional file 2A, B). The spectrum and intensity of emitted light was measured with the ILT950 spectrometer from International Light Technologies Inc. (Peabody, USA) whose detector was placed at the prospective position of the sample. The light intensity was given in irradiance (μW/cm^2^/s) and was converted to photon flux (photons/cm^2^/s). We attempted to match the total photon flux between light conditions. However, there is a relative lack of chromatic specificity of commercial green/yellow (500–590 nm) compared to shorter wavelength monochromatic LEDs [32], and so lower intensities were used in this assay to avoid chromatic overlap.

#### Shadow reflex assay

From 96 h prior to the assay, immature *Platynereis dumerilii* of comparable sizes (~20 mm in length) were separated into individual wells of six-well dishes (Greiner Bio One International GmbH) and starved of food for the remainder of the assay. At 48 h prior to the assay, the animals were placed in individual hemispherical concave wells (Additional file 1: Movie 1 and Additional file 2B) (diameter = 35 mm, depth = 15 mm) of a custom-made 36-well clear plastic plate and kept under normal culture conditions to allow novel tube formation. Upon introduction to the behavioural chamber (Additional file 2A, B) at ZT5 on the day of assay, the wells were refilled with fresh artificial seawater (ASW) before the assay. The assay was repeated for all six different light conditions (Additional file 2C, D) on each cohort of animals. The order in which the light conditions were presented was randomised for each new cohort of animals. The assay began with a 60-min acclimatisation period of exposure to the given ON light stimulus. After 45 min, each well was spiked with 50 μL of spinach-conditioned ASW to encourage foraging behaviour and make grading of the shadow response more apparent. For the shadow light regime, 12 shadow stimuli, each consisting of 2 s of sudden complete darkness (OFF), were given. These shadows were each separated by 60 s of normal (ON) light conditions to avoid desensitisation to the stimuli (Fig. 1a). Video recording of worm behaviour in both ON and OFF light conditions was facilitated as described previously [16], using an infrared (IR) (λ = 990 nm) LED array (Roschwege GmbH) illuminating the behavioural chamber and an IR high-pass filter restricting the video camera. Video was recorded continuously at 15 frames per second for the duration of the shadow stimuli.

#### Cirri removal

For the cirri dependence assay, size-matched immature wildtype sibling worms were collected. Half were randomly selected to undergo cirri removal and the other half were not. At 2 days prior to assay onset, all animals (both cut and uncut) were anaesthetised by submersion in an isotonic MgCl_2_/ASW solution (7.5% *w/v* in ddH_2_O mixed 1:1 with ASW). ‘Cut’ animals were placed dorsally on a glass cover slide and their two anal cirri and eight peristomal cirri were surgically removed using fine scalpel blades (Swann Morton Ltd.) and tungsten carbide needles (Fine Science Tools Ltd.) (adapted from [33]). Note that care was taken to avoid causing simultaneous damage to the palps or antennae (Additional file 4). Animals were then transferred to fresh ASW and allowed to recover for 1 h before being placed individually in hemispherical wells alongside their uncut siblings to prepare tubes for the shadow reflex assay. Upon starting the shadow assay 2 days after surgery, ‘cirri-removed’ animals had not begun to regrow their detached cirri (Additional file 4).

#### Whole mount *in situ* hybridisation

Expression of *Pdu-Go-opsin1* transcripts was localised in adult *Platynereis dumerilii* using an established whole mount *in situ* hybridisation protocol [34]. RNA probes were generated by in vitro transcription with T7 RNA Polymerase (New England Biolabs Inc. No. #M0251 L) incorporating digoxigenin-labelled UTPs (Roche Diagnostics GmbH, No. 11277073910). The probe template was generated by a PCR reaction conducted on immature wildtype *Platynereis dumerilii* cDNA using primers specific to the full coding sequences of the *Go-opsin1* gene (Additional file 5). Images of *Platynereis* heads stained by *in situ* hybridisation were taken using a Zeiss Axioplan2 light microscope. Overall head images were taken under a 10× and 20× air objective lens and cellular images with a 40× and 63× oil immersion objective.

#### *r-opsin1*^+^ cell-specific ablation

Transgenic *r-opsin1::eGFP-F2A-nitroreductase* animals were pre-screened for strong GFP fluorescence, along with the same number of size-matched wildtype animals, and were subjected to mtz (Sigma, catalogue no. M1547) treatment as described previously [24]. Mtz treatment took place for 3 days at a concentration of 12 mM mtz dissolved in 0.2% DMSO in ASW. Following treatment, animals were allowed to recover for 2 days in ASW before being placed in the shadow assay wells. Complete absence of GFP fluorescence was taken as an indicator of a successful ablation procedure. The shadow assay therefore took place 4 days after mtz treatment, well within the 7 day period found to preclude regrowth of the *r-opsin1*^+^ cells [24].

#### In vitro opsin absorption assay

The in vitro opsin absorption assay was conducted as described by Tsukamoto et al. [25]. Briefly, native *Platynereis c-opsin2* with the 1D4 tag (ETSQVAPA) on the C-termini was subcloned into the pMT vector. The construct was transiently expressed in mammalian COS-1 cells, incubated with 11-cis-retinal, and proteins were extracted with 1.25% DDM (Dojindo, Kumamoto, Japan), 20 mM HEPES, 140 mM NaCl, 0.25% cholesterol hemisuccinate (Sigma-Aldrich, St. Louis, MO, USA), 25 mM Tris and 10% glycerol at pH 7.0. The extract was mixed with 1D4-agarose overnight, and transferred to Bio-Spin columns (Bio-rad, Hercules, CA, USA). The columns were washed with 0.05% DDM, 2 mM ATP, 1 M NaCl, 3 mM MgCl_2_, 0.01% cholesterol hemisuccinate, 1 mM Tris and 10% glycerol in PBS, and subsequently washed with 0.05% DDM, 140 mM NaCl, 0.01% cholesterol hemisuccinate, 1 mM Tris, 10% glycerol and 20 mM HEPES at pH 7 (buffer A). The 1D4 tagged c-opsin2 was eluted with buffer A containing 0.45 mg/mL of 1D4 peptide (TETSQVAPA) (TOYOBO, Osaka, Japan). Absorption spectra of purified *Platynereis* c-opsin2 were recorded with a Shimadzu UV-2450 spectrophotometer (Shimadzu, Kyoto, Japan). During the spectral measurement, the samples were kept at 10 °C.

#### Phylogeny

The sequence alignment and tree calculation was performed using CLC Main Workbench, version 7.7.1. The alignment settings were as follows: gap open costs 10, gap extension costs 1, end gap costs as any other, very accurate alignment; settings tree: method UPGMA, protein distance measure: Kimura protein, 1000 bootstraps. The dataset supporting this tree is included in Additional file 6, as well as accession numbers and source organisms of protein sequences used.

#### Quantification and statistical analysis

Following assay completion, behaviour was analysed manually and individual animals were scored binarily (1 = withdrawal reflex, 0 = no reaction) for each shadow stimulus given. The withdrawal reflex is specifically defined as a shortening of total body length via retraction of the anterior and/or posterior end. If the animal is in the process of undulation associated with swimming or passive ventilation, this reflex can behaviourally appear as a sudden straightening of the body (Additional file 1: Movie 1). Prior to the assay, animals were placed in wells at random by a colleague with genotypes hidden from the behavioural analyser, and were revealed only once scoring had been conducted blindly. Individual SRSRs were then calculated according to the equation below:

$$ Shadow\ Reflex\ Success\ Rate\ (SRSR)=\frac{\# of\ successful\ shadow\ responses}{total\# of\ shadow s\ given} $$

Due to the fact that the number of successful shadow responses is always an integer value, SRSRs are an inherently discrete variable and thus the data takes on a stratified appearance. Statistical analysis and construction of box and whisker plots were conducted using Graphpad Prism 7.03 [35], where boxes represent interquartile range. Individual biological replicates are shown as diamonds in all cases. Datasets are approximately continuous (with 12 potential discrete values), unpaired and similarly distributed. Accordingly, significant differences between groups were calculated using two-tailed Mann–Whitney U tests with a *P* value threshold of 0.05. The Benjamini–Hochburg correction for multiple testing was applied to these tests in Fig. 1b, where each dataset was tested twice. For Fig. 3b, the lack of statistical significance between groups was calculated by assessment of whether the difference between pseudo-medians, calculated using Hodges–Lehmann non-parametric estimator, exceeds 95% confidence intervals. Under any single condition in every figure, SRSRs were pooled from at least three separate independent experiments. Precise values of *n* and the statistical parameters used in each figure are provided in the accompanying figure legends.

## Additional files

Additional file 2: Hardware setup of shadow reflex assay. (A) Image of lid of behavioural chamber taken from the perspective of the subject with 520 nm light array switched on. Other wavelength arrays can be seen switched off beneath it. (B) Image of occupied hemispherical 36-well behaviour plate placed in position under 520 nm light. Adult *Platynereis* worms can be seen attached to the bottom of these wells ready for the shadow reflex assay to be conducted. (C) Photon irradiance (photons/cm^2^/s) spectra and (D) total photon irradiance (photons/cm^2^/s) (AUC) of each light stimulus used in the shadow reflex assay. The same white light stimulus is shown in both graphs to illustrate the intensity disparity between 400 and 470 nm and 500–590 nm stimuli. (TIFF 6563 kb)

Additional file 3: *Go-opsin1* mutant lunar phase maturation comparison. Number of animals undergoing maturation per lunar day normalised to the total number of worms of that genotype under 500 nm monochromatic solar and lunar stimuli between sibling animals collected over the course of 12 consecutive months (August 2016 to August 2017). (TIFF 307 kb)

Additional file 4: Surgical cirri removal. Differential interference contrast image of *Platynereis* head 2 days after cirri removal surgery, 10× and 40× magnification. Surgical cutting points are denoted by dotted lines. The adult eyes (ae), palps (p) and antennae (a) are also labelled. (TIFF 5841 kb)

Additional file 5: **Table S1.** Go-opsin1 primer sequences for genotyping and WMISH probe generation. (PDF 28 kb)

Additional file 6: Accession numbers and source organisms of protein sequences used in phylogenetic tree. (PDF 39 kb)

## Abbreviations

ASW: artificial seawater
mtz: metronidazole
*Pdu*: *Platynereis dumerilii*
SRSR: shadow response success rate
