# Supplementary figures and images for: A Go-type opsin mediates the shadow reflex in the annelid Platynereis dumerilii
Source: BMC Biol. 2018 Apr 18;16:41. doi: 10.1186/s12915-018-0505-8 (PMC5904973; doi:10.1186/s12915-018-0505-8)

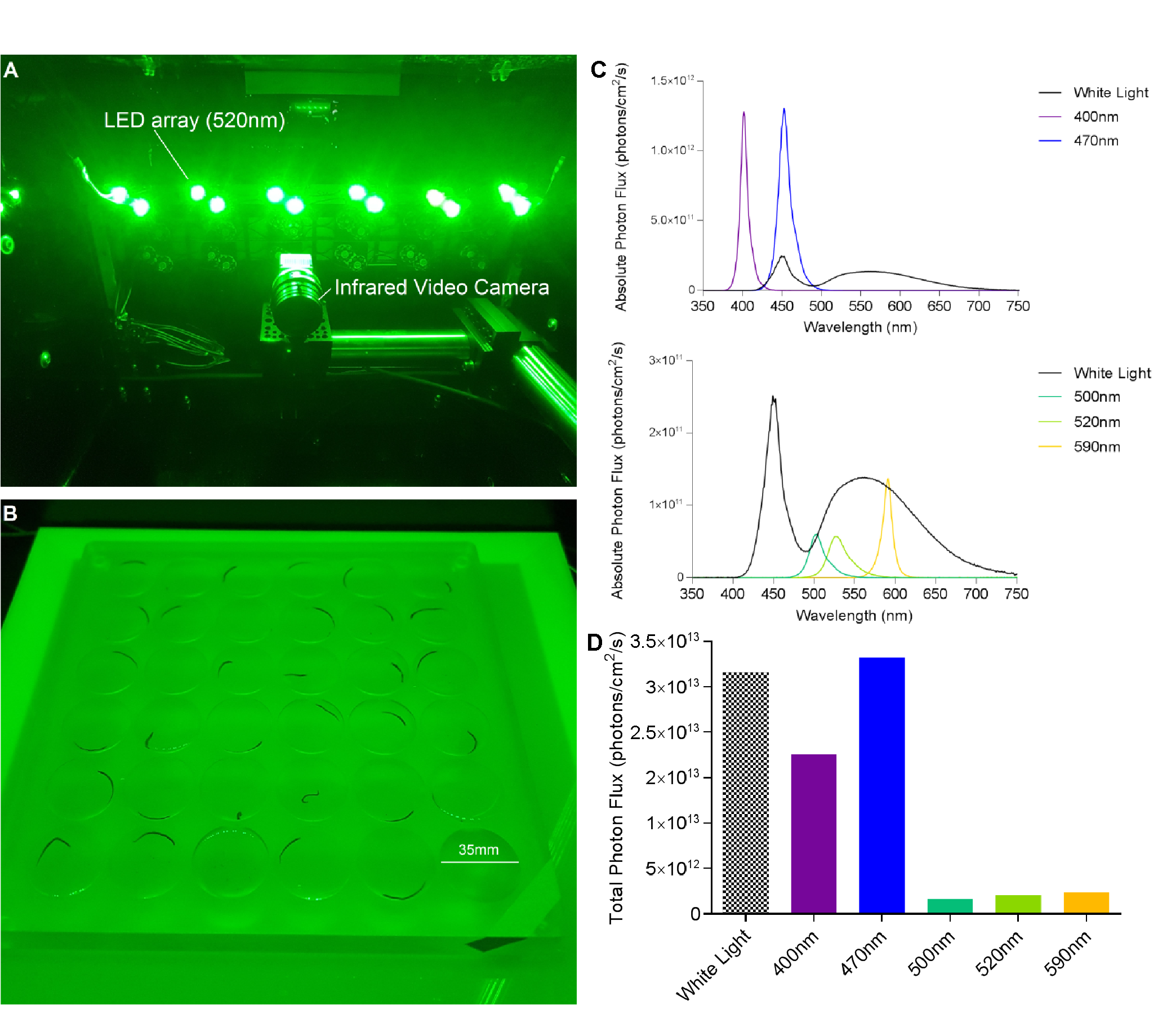

Supplement: Supplementary file 2 — Hardware setup of shadow reflex assay. (A) Image of lid of behavioural chamber taken from the perspective of the subject with 520 nm light array switched on. Other wavelength arrays can be seen switched off beneath it. (B) Image of occupied hemispherical 36-well behaviour plate placed in position under 520 nm light. Adult Platynereis worms can be seen attached to the bottom of these wells ready for the shadow reflex assay to be conducted. (C) Photon irradiance (photons/cm2/s) spectra and (D) total photon irradiance (photons/cm2/s) (AUC) of each light stimulus used in the shadow reflex assay. The same white light stimulus is shown in both graphs to illustrate the intensity disparity between 400 and 470 nm and 500–590 nm stimuli. (TIFF 6563 kb) [file 12915_2018_505_MOESM2_ESM.tif]

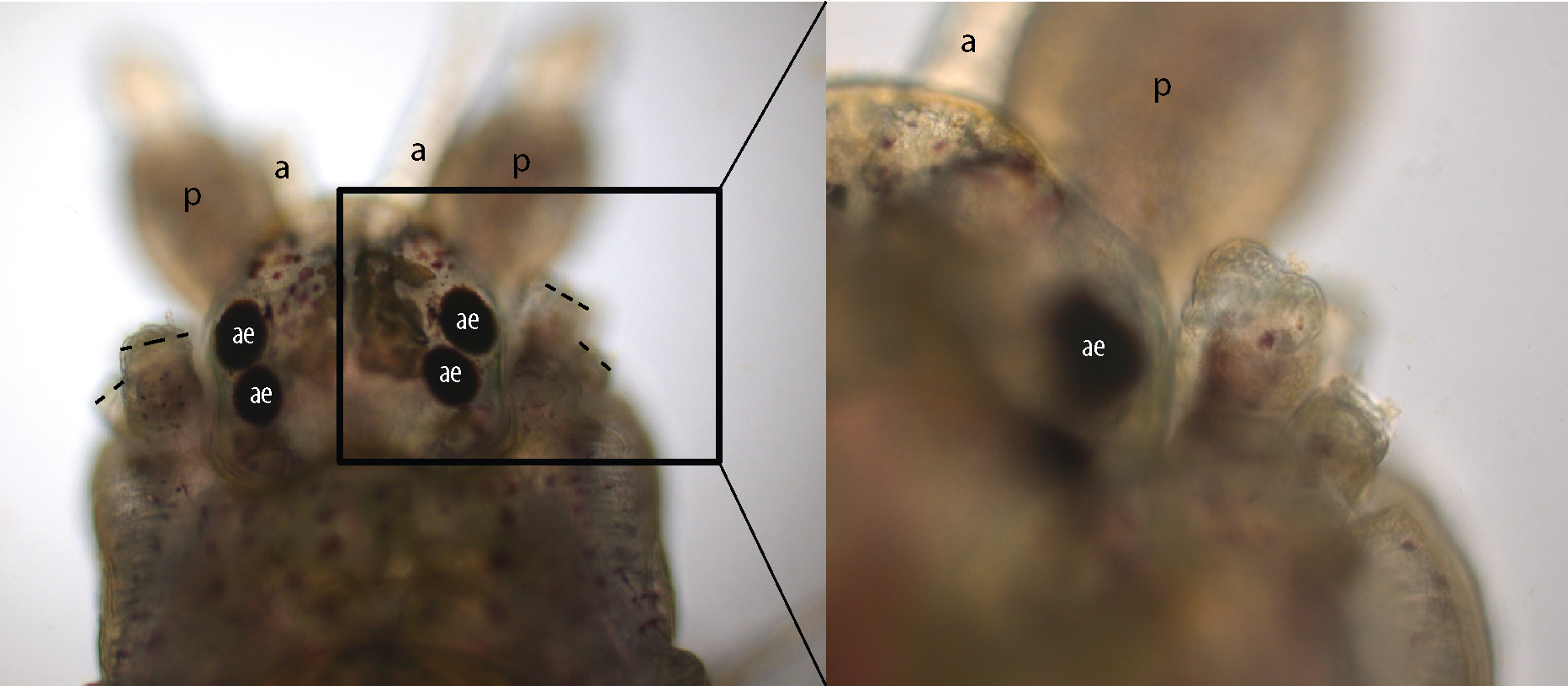

Supplement: Supplementary file 4 — Surgical cirri removal. Differential interference contrast image of Platynereis head 2 days after cirri removal surgery, 10× and 40× magnification. Surgical cutting points are denoted by dotted lines. The adult eyes (ae), palps (p) and antennae (a) are also labelled. (TIFF 5841 kb) [file 12915_2018_505_MOESM4_ESM.tif]
