# Supplementary material for: A Go-type opsin mediates the shadow reflex in the annelid Platynereis dumerilii
Source: BMC Biol. 2018 Apr 18;16:41. doi: 10.1186/s12915-018-0505-8 (PMC5904973; doi:10.1186/s12915-018-0505-8)
Supplement: Supplementary file 5 — Table S1. Go-opsin1 primer sequences for genotyping and WMISH probe generation. (PDF 28 kb) [file 12915_2018_505_MOESM5_ESM.pdf]

**(A)** *Pdu-Go-opsin1* exon 1 primer sequences used for genotyping	
Go-Ops_exon1_Fwd	CTGCTGAATGCCATTAGTTGACGG
Go-Ops_exon1_Rev	CTGCTGAATGCCATTAGTTGACGG
**(B)** *Pdu-Go-opsin1* primer sequences used to extract full *Go-opsin1* coding sequence from cDNA to prepare Whole Mount *In situ* Hybridisation RNA probes	
Go-ops_fullCDS_Fwd	CTGCTGAATGCCATTAGTTGACGG
Go-ops_fullCDS_Rev	CAAGTCACAAGTGCTCACATGTAT
