# Supplementary material for: A Go-type opsin mediates the shadow reflex in the annelid Platynereis dumerilii
Source: BMC Biol. 2018 Apr 18;16:41. doi: 10.1186/s12915-018-0505-8 (PMC5904973; doi:10.1186/s12915-018-0505-8)
Supplement: Supplementary file 6 — Accession numbers and source organisms of protein sequences used in phylogenetic tree. (PDF 39 kb) [file 12915_2018_505_MOESM6_ESM.pdf]

**Species name**	**Protein name**	**Accession Number**
Anopheles gambiae	GPRopsin11	EAA07662.5
Anopheles gambiae	GPRopsin12	EAA08098.2
Daphnia pulex	Pteropsin3	EFX87346.1
Daphnia pulex	Pteropsin1	EFX87345.1
Daphnia pulex	Pteropsin4	EFX86931.1
Daphnia pulex	Pteropsin7	EFX80367.1
Daphnia pulex	Pteropsin8	EFX80369.1
Platynereis dumerilii	c-Opsin1	AAV63834.1
Takifugu rubripes	TMT Opsin	AAL83430.1
Homo sapiens	Encephalopsin	AAD32671.1
Mus musculus	Encephalopsin/Opsin-3	AAI05920.1
Petromyzon marinus	Pineal Opsin	O42490.1
Danio rerio	VAL Opsin	NP_571661.1
Salmo salar	Vertebrate Ancient Opsin	NP_001117098.1
Columba livia	Blue-sensitive Opsin	NP_001296977.1
Lethenteron camtschaticum	Rhodopsin	P22671.1
Petromyzon marinus	Rhodopsin	Q98980.1
Xenopus laevis	Rhodopsin	P29403.1
Homo sapiens	Rhodopsin	NP_000530.1
Gallus gallus	Rhodopsin	NP_001025777.1
Papilio glaucus	Rhodopsin	AAD34220.1
Schistocerca gregaria	Rhodopsin	Q94741.1
Apis mellifera	Rhodopsin	Q17053.1
Drosophila melanogaster	Rhodopsin6	XP_002031049.1
Drosophila melanogaster	Rhodopsin2	NP_524398.1
Drosophila melanogaster	Rhodopsin1	NP_524407.1
Drosophila melanogaster	Rhodopsin4	NP_476701.1
Drosophila melanogaster	Rhodopsin3	NP_524411.1
Drosophila melanogaster	Rhodopsin5	NP_477096.1
Daphnia pulex	UV-wavelength Opsin	EFX81332.1
Procambarus clarkii	Rhodopsin	P35356.1
Limulus polyphemus	Ocellar Opsin	NP_001301089.1
Mizuhopecten yessoensis	Rhodopsin	O15973.1
Platynereis dumerilii	Rhabdomeric Opsin	CAC86665.1
Enteroctopus dofleini	Rhodopsin	P09241.1
Todarodes pacificus	Rhodopsin	P31356.2
Sepia officinalis	Rhodopsin	O16005.1
Loligo forbesii	Rhodopsin	P24603.1
Xenopus laevis	Melanopsin	NP_001079143.1
Homo sapiens	Melanopsin	NP_150598.1
Mus musculus	Melanopsin	EDL24885.1
Bos taurus	RGR-Opsin	NP_786969.1
Homo sapiens	RGR-Opsin	NP_001012738.1
Mus musculus	RGR-Opsin	NP_067315.1
